# SYP-5 regulates meiotic thermotolerance in *Caenorhabditis elegans*

**DOI:** 10.1093/jmcb/mjab035

**Published:** 2021-06-03

**Authors:** Yuanyuan Liu, Qiuchen Zhao, Hui Nie, Fengguo Zhang, Tingting Fu, Zhenguo Zhang, Feifei Qi, Ruoxi Wang, Jun Zhou, Jinmin Gao

**Affiliations:** Institute of Biomedical Sciences, College of Life Sciences, Key Laboratory of Animal Resistance Biology of Shandong Province, Shandong Normal University, Jinan 250014, China

**Keywords:** meiosis, thermotolerance, synaptonemal complex, crossover regulation, *Caenorhabditis elegans*, SYP-5

## Abstract

Meiosis produces the haploid gametes required by all sexually reproducing organisms, occurring in specific temperature ranges in different organisms. However, how meiotic thermotolerance is regulated remains largely unknown. Using the model organism *Caenorhabditis elegans*, here, we identified the synaptonemal complex (SC) protein SYP-5 as a critical regulator of meiotic thermotolerance. *syp-5*-null mutants maintained a high percentage of viable progeny at 20°C but produced significantly fewer viable progeny at 25°C, a permissive temperature in wild-type worms. Cytological analysis of meiotic events in the mutants revealed that while SC assembly and disassembly, as well as DNA double-strand break repair kinetics, were not affected by the elevated temperature, crossover designation, and bivalent formation were significantly affected. More severe homolog segregation errors were also observed at elevated temperature. A temperature switching assay revealed that late meiotic prophase events were not temperature-sensitive and that meiotic defects during pachytene stage were responsible for the reduced viability of *syp-5* mutants at the elevated temperature. Moreover, SC polycomplex formation and hexanediol sensitivity analysis suggested that SYP-5 was required for the normal properties of the SC, and charge-interacting elements in SC components were involved in regulating meiotic thermotolerance. Together, these findings provide a novel molecular mechanism for meiotic thermotolerance regulation.

## Introduction

Meiosis is a specialized cell division required by all types of sexually reproducing organisms to produce haploid gametes from diploid germ cells. A single round of DNA replication is followed by two consecutive cell divisions (meiosis I and II) to reduce the chromosome number. A series of meiosis-specific events take place to ensure accurate chromosome segregation during this process, including homologous chromosome pairing, formation of the synaptonemal complex (SC) between paired homologs, interhomolog recombination, and crossover (CO) formation. Along with sister chromatid cohesion, COs provide physical attachments (chiasmata) between homologs, enabling their proper alignment at the metaphase plate and accurate segregation at meiosis I. Meiotic recombination plays a crucial role in the generation of new variation, especially in plants. Meiotic recombination shuffles genetic information during gamete production, and is thus an important factor in inheritance, adaptation, and responses to selection. In humans, reduced/altered meiotic recombination is associated with a higher incidence of trisomies (Hassold and Sherman, 2000).

Two parallel processes, synapsis and homologous recombination, are required during meiotic prophase to establish COs between homologs and thus accurate meiotic chromosome segregation. Meiotic recombination is initiated by the conserved topoisomerase VI-like SPO11-induced double-strand breaks (DSBs) (Keeney et al., 1997). Following DSB formation, the 5ʹ ends are resected, and recombinant proteins (RPA1, RAD51, DMC1) are sequentially recruited to the 3ʹ single-strand tails to mediate strand invasion and form meiotic recombination intermediates (Neale and Keeney, 2006). The intermediates can be resolved via two pathways: the CO formation DSB repair pathway, and the noncrossover (NCO) formation synthesis-dependent strand-annealing pathway (Sung and Klein, 2006). In many organisms, CO formation can be subdivided into the Zip−Mer−Msh (ZMM)-mediated pathway, which is subjected to interference (type I), and the MUS81-mediated pathway, which is insensitive to interference (type II) (Gray and Cohen, 2016; Saito and Colaiácovo, 2017).

Meiosis, especially CO regulation, can be significantly affected by external and intrinsic factors, the latter including age and sex, which can affect meiotic recombination across species including humans (Rose and Baillie, 1979; Francis et al., 2007; Hassold et al., 2007). A well-known external factor affecting meiosis is altered temperature. A suboptimal temperature affects CO frequency and distribution, while extreme temperatures disrupt core structures, the axis, and the SC (Morgan et al., 2017). Meiosis-permissive temperature ranges vary dramatically among organisms (Bomblies et al., 2015). In plants, some organisms have optimal meiosis temperature below 15°C (e.g. *Hyacinthus orientalis* and *Endymion nonscriptus*), while some have optimal meiosis temperatures of 20°C or higher (e.g. barley and wheat). In animals, mammals have an optimal meiosis temperature in a narrow range around 30°C, while meiosis in *Caenorhabditis elegans* is normal within a wider range from 12°C to 26°C (Hirsh et al., 1976).

While well described, the molecular mechanisms underlying the regulation of meiotic thermotolerance are largely unknown. Only a handful of genes have been reported to be involved in meiotic thermotolerance across organisms, including *ZIP1* in budding yeast (Chan et al., 2009), the cyclin-dependent kinase *CDKG1* in *Arabidopsis* (Zheng et al., 2014), the meiotic recombination gene *Dmc1* in wheat (Draeger et al., 2020), and *ife-2*, *pgl-1*, and *pch-2* in *C. elegans* (Song et al., 2010; Bilgir et al., 2013; Deshong et al., 2014). Moreover, how these genes regulate meiotic thermotolerance still needs to be explored.

Here, we analyzed three groups of meiotic mutants that are potentially sensitive to the elevated temperature in *C. elegans*. We report that the SC central region protein SYP-5 plays a critical role in regulating meiotic thermotolerance. Mutation of *syp-5* reduces progeny viability at elevated temperatures. Cytological analysis also reveals significant impacts of temperature on CO regulation and bivalent formation in this mutant. Temperature switching assays consistently suggest that meiotic events during pachytene are sensitive to elevated temperature. Moreover, we provide evidences that SYP-5 is required for the normal properties of the SC, and that charge-interacting elements (CIEs) within SYP-5 and SYP-4 are critical for meiotic thermotolerance at elevated temperatures. Further analysis of human variants reveals the presence of mutations that can affect CIE formation within SC components, providing an interesting direction for future translational study. Our findings define new molecular mechanisms underlying meiotic thermotolerance, providing insights into meiosis evolution and translational relevance for breeding and human reproductive health.

## Results

### Plate phenotypes of syp-5 mutants are temperature-sensitive

**Figure 1:**
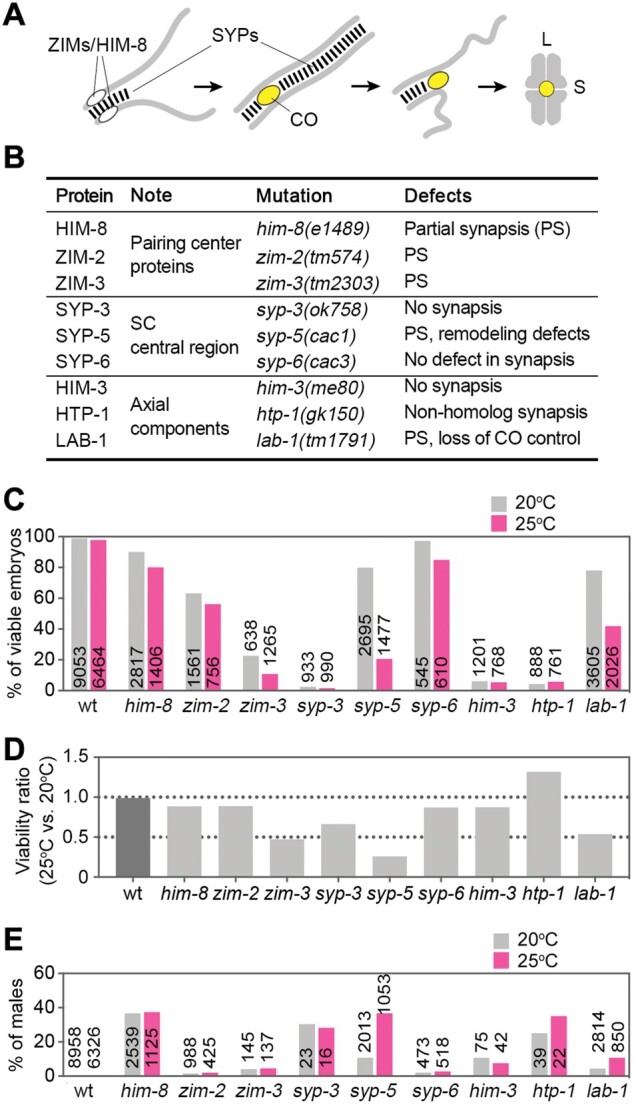
Screening for meiotic mutants that are sensitive to the elevated temperature. (**A**) Cartoon depicting meiotic chromosome dynamics in *C. elegans*. Localization of meiotic proteins are indicated. CO, crossover; L, bivalent long-arm; S, bivalent short arm. (**B**) A list of mutants used for temperature sensitivity screening. PS, partial synapsis. (**C**) Percentage of viable embryos at the standard (20°C) and high (25°C) culture temperatures. (**D**) Viability ratio between 25°C and 20°C culture conditions. (**E**) Percentage of male progeny at the standard (20°C) and high (25°C) culture temperatures.

To identify factors affecting meiotic thermotolerance, we analyzed three groups of *C. elegans* mutants that affect various meiotic events (Figure 1A and B). The first group included mutants that have lost functional pairing center proteins and thus abolish the pairing of a subset of chromosomes during meiotic prophase (*him-8*, *zim-2*, and *zim-3*) (Phillips et al., 2005; Phillips and Dernburg, 2006). The second group included mutants missing various SC central region components, including *syp-3*, *syp-5*, and *syp-6* (Smolikov et al., 2007; Hurlock et al., 2020; Zhang et al., 2020). The third group included mutations missing SC lateral/axial components (*him-3*, *htp-1*, and *lab-1*) (Zetka et al., 1999; Couteau and Zetka, 2005; Martinez-Perez and Villeneuve, 2005; de Carvalho et al., 2008).

We first compared the percentages of viable embryos under normal culture temperature (20°C) and high-temperature (25°C) conditions. Consistent with previous findings, the mutants exhibited various levels of embryonic lethality (Figure 1C; Zetka et al., 1999; Couteau and Zetka, 2005; Martinez-Perez and Villeneuve, 2005; Phillips et al., 2005; Phillips and Dernburg, 2006; Smolikov et al., 2007; Zhang et al., 2020). Interestingly, at the elevated temperature, the percentage of viable embryos was further reduced in some mutants, including *zim-3*, *syp-5*, and *lab-1* mutants (Figure 1D), suggesting that these meiosis-specific components may be required for thermotolerance of meiosis in *C. elegans*. Of these temperature-sensitive mutants, *syp-5* mutants exhibited the greatest effect, with 80% embryo viability at 20°C reducing to 20% at 25°C.

While embryonic lethality may be associated with errors in meiotic chromosome segregation and defects in embryonic development in *C. elegans*, a high incidence of male progeny (Him) is indicative of meiotic chromosome segregation errors. By analyzing male progeny of the meiotic mutants, we observed a significantly increased percentage of male progeny in *syp-5* mutants at 25°C (37%) compared to that at 20°C (11%) (Figure 1E), suggesting that meiotic chromosome segregation errors are exacerbated by high temperature. This high Him phenotype is also consistent with our previous finding that X chromosome synapsis is frequently affected in *syp-5* mutants (Zhang et al., 2020).

### Defects in SC assembly and disassembly are not temperature-sensitive

**Figure 2:**
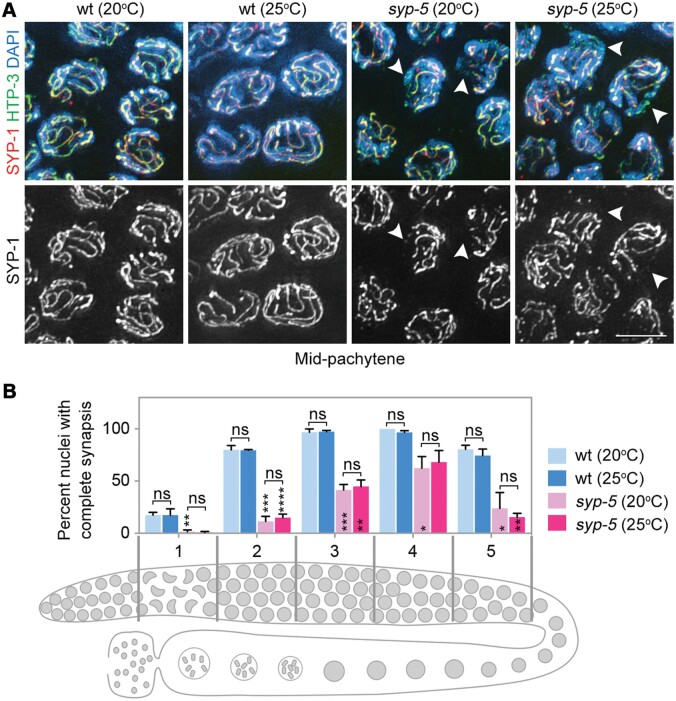
Defects in SC assembly are not temperature-sensitive in *syp-5* mutants. (**A**) Images of mid-pachytene nuclei in the wild-type and *syp-5* mutants stained to visualize HTP-3 (green), SYP-1 (red), and DNA (blue). Arrowheads indicate chromosomes with synapsis defects. Scale bar, 5 μm. (**B**) Kinetics of meiotic chromosome synapsis in the wild-type and *syp-5* mutants. The numbers on the x-axis correspond to regions of the gonad depicted in the cartoon at the bottom. Meiotic progression is from left to right. Error bars represent 95% confidence intervals. Asterisks indicate statistical significance between different genotypes under the same condition: *P < 0.05, **P < 0.01, ***P < 0.001, ****P < 0.0001, by the two-tailed unpaired *t*-test. ns, not statistically significant.

Our lab and others recently showed that SYP-5 and SYP-6 are structural components of the SC, and they play redundant but not identical roles in SC assembly and CO regulation (Hurlock et al., 2020; Zhang et al., 2020). SYP-5 and SYP-6 show distinct expression patterns, and the most abundant chromosome association of SYP-5 presents in late pachytene stage (Supplementary Figure S1; Zhang et al., 2020). In *syp-5* mutants, SYP-6 is present to support synapsis and CO formation, and synapsis defects were observed on a subset of meiotic chromosomes during early meiotic prophase. To understand whether the elevated embryonic lethality was associated with synapsis defects at elevated temperatures in *syp-5* mutants, we examined chromosome synapsis by coimmunostaining the axial component HTP-3 and the central region protein SYP-1 (Figure 2A). The kinetics of chromosome synapsis were not affected by the elevated temperature in wild-type worms. Consistent with our previous finding, asynapsis of a subset of chromosomes was frequently observed during early meiotic prophase in *syp-5* mutants, but the asynapsis frequency and kinetics were not altered by the elevated temperature, suggesting that SC assembly defects are not significantly affected by the elevated temperature (25°C) (Figure 2B).

Another synapsis defect in *syp-5* mutants is premature SC disassembly upon pachytene exit (Zhang et al., 2020). However, no change in SC disassembly kinetics was observed upon pachytene exit at the elevated temperature (Supplementary Figure S2). Therefore, SC assembly and disassembly in *syp-5* mutants are not temperature-sensitive at the examined temperatures.

### Overall kinetics of RAD-51 foci are not altered by the elevated temperature

To understand how meiotic recombination was affected by temperature in *syp-5* mutants, we examined focus formation of the recombination protein RAD-51 in the germline. RAD-51 is a protein required for strand invasion/exchange during DNA DSB repair and associates with DSB repair sites (Supplementary Figure S3A; Sung, 1994; Colaiácovo et al., 2003); the kinetics of RAD-51 foci in the germline thus represent the kinetics of DSB repair. The kinetics of RAD-51 foci in wild-type gonads were not affected by the elevated temperature. In *syp-5* mutants, RAD-51 foci increased during mid-to-late pachytene stages compared to that in the wild-type. However, the overall kinetics were not significantly affected by the elevated temperature, although reduced RAD-51 focus formation was observed during late pachytene stage, which may suggest faster repair (Supplementary Figure S3B). No RAD-51 foci were observed in diplotene nuclei in *syp-5* mutants at both temperatures (data not shown). These observations suggest that although DSB formation/repair kinetics are affected by *syp-5* mutation, the early meiotic recombination events are not likely significantly affected by the elevated temperature.

### CO designation is delayed and dysregulated by the elevated temperature in syp-5 mutants

**Figure 3:**
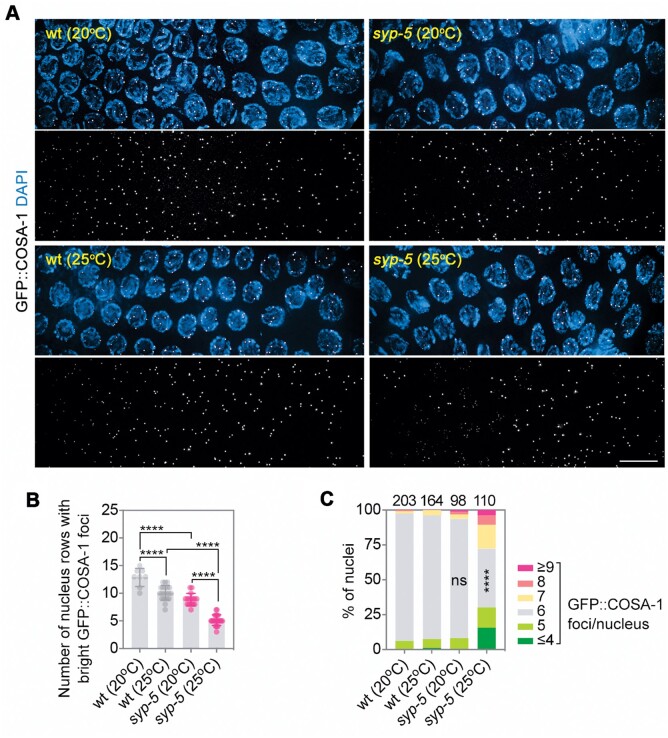
Analysis of CO designation at different temperatures. (**A**) GFP::COSA-1 (green) focus formation in mid-to-late pachytene nuclei of wild-type and *syp-5* mutant worms grown at 20°C or 25°C. Chromatin was stained with DAPI (blue). Scale bar, 10 μm. (**B**) Quantification of row numbers of pachytene nuclei containing bright GFP::COSA-1 foci in wild-type or *syp-5* mutant background at different temperatures. ****P < 0.0001, by the two-tailed unpaired *t*-test. (**C**) Quantification of GFP::COSA-1 focus numbers in late pachytene nuclei of worms grown at different temperatures. Asterisks indicate statistical significance between different genotypes under the same condition: ****P < 0.0001, by Chi-square test. ns, not statistically significant.

Although DSB formation and repair kinetics are affected in *syp-5* mutants, CO designation during late pachytene stage is not significantly affected, as examined by GFP::COSA-1 focus formation (Zhang et al., 2020). To analyze this process in detail, we counted the number of nucleus rows with bright GFP::COSA-1 foci corresponding to sites designated for CO formation during pachytene stage (Yokoo et al., 2012). In the wild-type background, elevated temperature slightly reduced the number of nucleus rows containing bright GFP::COSA-1 foci (13 vs. 10). At the normal temperature, *syp-5* mutants also exhibited reduced row numbers compared to the wild-type (13 vs. 9), while at the elevated temperature, GFP::COSA-1 row numbers were significantly reduced in *syp-5* mutants compared to the wild-type (10 vs. 5) (Figure 3A and B). These observations suggest that the timing of CO designation is delayed by the elevated temperature in *syp-5* mutants.

In addition to the delayed designation, CO formation is dysregulated in *syp-5* mutants at high temperature. In the wild-type background, six COSA-1 foci were observed in most of the nuclei during late pachytene at both temperatures. In the *syp-5* mutant background, while six COSA-1 foci were observed at the normal temperature, both increased and reduced COSA-1 foci were observed at the higher temperature (Figure 3C), suggesting abnormal regulation of CO formation at the elevated temperature even though SC assembly and disassembly were not affected.

### Univalent formation is elevated in syp-5 mutants at a high temperature

**Figure 4:**
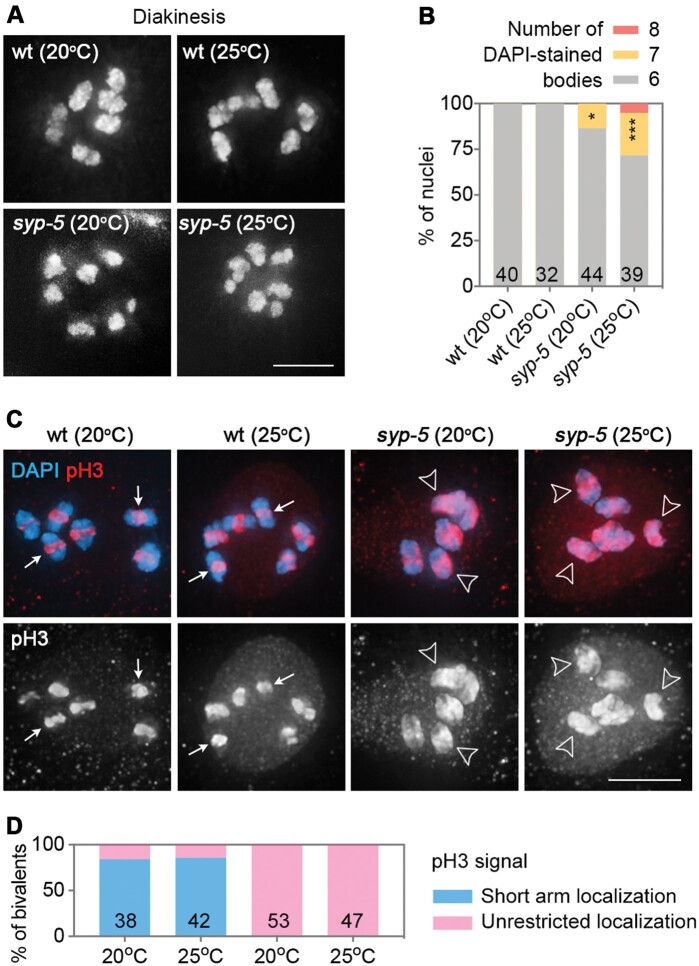
High temperature impairs bivalent formation in *syp-5* mutants. (**A**) Images of DAPI-stained bodies in −1 oocytes at diakinesis in the indicated genotypes and temperatures. Scale bar, 5 μm. (**B**) Quantification of DAPI-stained bodies in −1 oocytes in the indicated genotypes and temperatures. Numbers of oocytes scored are indicated. Asterisks indicate statistical significance between different genotypes under the same condition: *P < 0.05, ***P < 0.001, by Chi-square test. (**C**) Images of −1 oocytes stained for pH3 (red) and DAPI (blue). White arrows indicate short arm localization of pH3, and open arrowheads indicate an unrestricted localization of pH3. Scale bar, 5 μm. (**D**) Quantification of bivalents with different pH3 localization patterns in −1 oocytes in the indicated genotypes and temperatures. Numbers of bivalents analyzed are indicated.

Wild-type diakinesis oocytes contained six DAPI-stained bodies, corresponding to six pairs of attached homologous chromosomes (bivalents), and bivalent formation was not affected at the examined temperatures (Figure 4A). In *syp-5* mutants, ∼86% of diakinesis nuclei contained six DAPI-stained bodies, with the remaining nuclei (∼14%) containing seven DAPI-stained bodies (Figure 4B), suggesting a lack of COs between one pair of homologous chromosomes in those nuclei. At the higher temperature, a higher proportion of diakinesis nuclei contained more than six DAPI-stained bodies (∼28%) (Figure 4B), suggesting that high temperature aggravates univalent formation in *syp-5* mutants. Defects in bivalent formation in diakinesis are consistent with the abnormal COSA-1 foci formation observed during late pachytene stage in *syp-5* mutants at the higher temperature.

However, of note, the lack of CO formation is not likely to be the only cause of high embryonic lethality in *syp-5* mutants at higher temperatures, given that ∼72% oocytes contain the normal number of bivalents and the mutants have only ∼20% viable progeny. Another contribution of the high embryonic lethality in *syp-5* mutants could be the extra COs formed at a high temperature, where ∼34% of the nuclei contain more than six GFP::COSA-1 foci. Indeed, the association between excess COs and elevated embryonic lethality in *C. elegans* is supported by a recent work (Hollis et al., 2020).

Moreover, defects in chromosome remodeling in *syp-5* mutants can also contribute to the high embryonic lethality (Figure 4C and D). At the end of meiotic prophase, chromosome remodeling results in histone H3 phosphorylation in the bivalent short arm region, where inter-sister cohesin is also phosphorylated for cleavage during meiosis I (Ferrandiz et al., 2018). By immunostaining Ser10 phosphorylated H3 (pH3) in late prophase oocytes, we found that this histone modification was restricted to the bivalent short arms in the wild-type at both temperatures. However, in *syp-5* mutants, pH3 spread throughout the whole bivalent at both temperatures (Figure 4C and D), suggesting a defect in chromosome remodeling in *syp-5* mutants. In conclusion, multiple factors including abnormal CO formation and defects in chromosome remodeling together cause the high embryonic lethality in *syp-5* mutants at the elevated temperature.

### Elevated frequency of meiotic chromosome segregation errors in syp-5 mutants at a higher temperature

**Figure 5:**
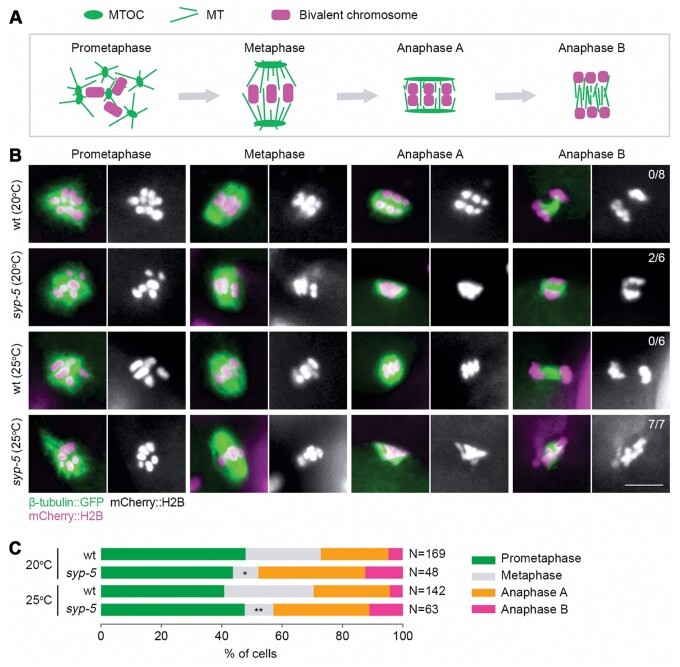
Defects in meiotic chromosome segregation in *syp-5* mutants at a high temperature. (**A**) Cartoon depicting the assembly of the meiotic spindle and its morphological changes during meiosis I in *C. elegans*. MTOC, microtubule-organizing center; MT, microtubule. (**B**) Meiotic chromosome segregation defects in *syp-5* mutants. *syp-5* mutants were crossed with worms expressing GFP-tagged tubulin (green) and mCherry-tagged histone H2B (magenta). Young adult worms (24 h post-L4) were fixed and the meiotic spindle was examined. Frequencies of dividing cells with anaphase chromosome segregation errors are indicated. Scale bar, 5 μm. (**C**) Percentages of cells during meiosis I at the indicated stages. Numbers of cells scored are indicated. Asterisks indicate statistical significance between different genotypes under the same condition: *P < 0.05, **P < 0.01, by the two-sided Fisher’s exact test.

To confirm whether elevated temperature in *syp-5* mutants is indeed associated with meiotic chromosome segregation errors, *syp-5* mutants were crossed with worms expressing H2B::mCherry and β-tubulin::GFP (FM125 strain) to visualize the meiotic spindle (Figure 5A and B). The FM125 strain showed only a minor reduction (∼10%) in viability at 25°C compared to that at 20°C. However, *syp-5* mutation resulted in a significant reduction in viable progeny in this strain at the higher temperature (∼90% reduction), consistent with our finding that SYP-5 is critical for meiosis thermotolerance (Figure 1).

Abnormal bivalent remodeling during late meiotic prophase does not cause the premature dissociation of homologs or sister chromatids during meiosis I, since six DAPI-stained bodies were still observed in most of the prometaphase cells in *syp-5* mutants (Figure 5). However, during anaphase I, chromosome lagging was observed in *syp-5* mutants but not in the control strain, and this error was more frequent at the higher temperature (Figure 5B). By quantifying meiotically dividing cells at different stages, we observed a significant reduction of cells at metaphase I stage in *syp-5* mutants compared to that in the wild-type (Figure 5C), which may suggest defects in meiotic chromosome alignment. These observations suggest that elevated temperature indeed causes meiotic chromosome segregation errors in *syp-5* mutants, consistent with the elevated embryonic lethality and Him seen at this temperature.

### A temperature switching assay suggests that meiotic events before pachytene exit are temperature-sensitive in syp-5 mutants

**Figure 6:**
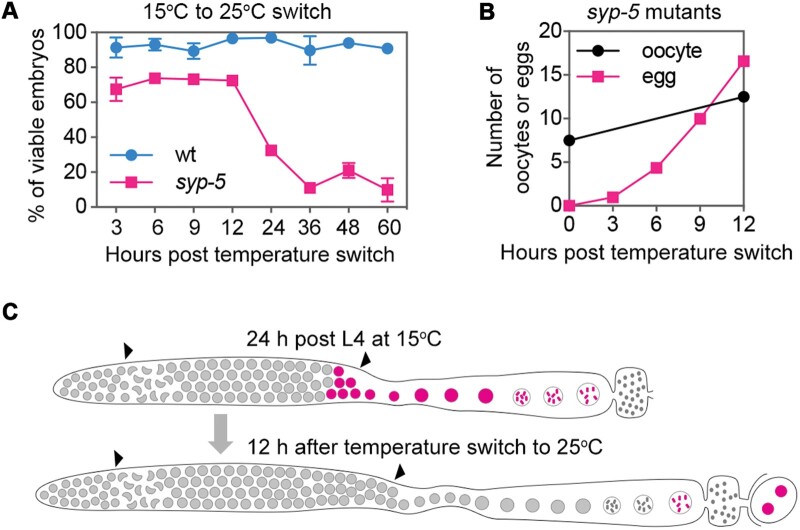
A temperature switching assay reveals meiotic stages sensitive to the elevated temperature. (**A**) Analysis of embryonic viability after temperature switching from 15°C to 25°C. (**B**) Quantification of oocyte number (diplotene and diakinesis cells) in the germline and accumulated eggs laid by *syp-5* mutants at the indicated time points. (**C**) A cartoon depicts the progression of germ cells 12 h after temperature switching. Nuclei progressing through meiosis with unaffected viability are depicted in red.

To further clarify which meiotic defects accounted for the high embryonic lethality in *syp-5* mutants at the higher temperature, we performed a temperature switching assay. In this assay, worms were grown at low temperature (15°C) until maturation into young adults (24 h post-L4) before being switched to a high temperature condition (25°C). Analyzing the viable progeny after the temperature switch, there was no apparent change of viable embryos in wild-type worms. Interestingly, there was also no significant change in progeny viability for *syp-5* mutants within the first 12 h after temperature switching. However, 12‒36 h after temperature switching, there was a dramatic reduction in progeny viability from ∼75% to ∼15% in the mutants (Figure 6A). These observations suggest that oocytes laid during the early time point are not significantly affected by temperature.

To further understand which meiotic stages were affected by temperature in *syp-5* mutants, we counted oocyte number in the gonads and eggs laid after the temperature switch. Comparing these numbers would allow us to estimate the earliest germ cell population affected by the elevated temperature. Cytological analysis showed that the oocyte number (including diplotene and diakinesis nuclei) increased from 7.5 per gonad arm to 12.5 per gonad arm, and ∼17 eggs were laid from each gonad arm within the first 12 h after temperature switching (Figure 6B). These laid eggs were thus derived from a cell population including all the late prophase oocytes and a subset of late pachytene cells in the worms upon the temperature switch (Figure 6C). Given that these laid eggs did not exhibit elevated embryonic lethality (Figure 6A), meiotic events before pachytene exit are likely to be sensitive to the elevated temperature in *syp-5* mutants, consistent with the idea that abnormal CO regulation during pachytene at the higher temperature causes high embryonic lethality in this mutant background.

### SYP-5 is required for the normal properties of the SC

**Figure 7:**
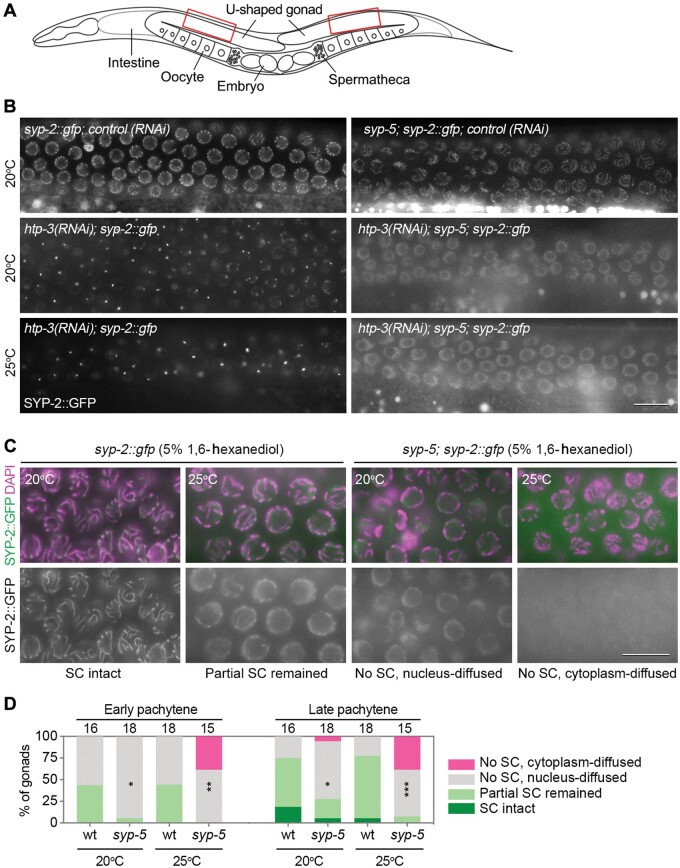
SYP-5 is required for the normal properties of the SC. (**A**) A cartoon depicts the organization of the germlines in an adult hermaphrodite. The red box regions correspond to cells at early‒mid-pachytene stages. (**B**) *htp-3(RNAi)* by feeding results in SC polycomplex formation in *syp-2::gfp* worms, but not in *syp-5; syp-2::gfp* worms. Images were captured in live animals at the boxed regions indicated in **A**. Scale bar, 10 μm. (**C**) Gonads dissected from worms of the indicated genotypes and culture temperatures were treated with 5% 1,6-hexanediol, then fixed, and stained with DAPI (magenta). Images show examples of different SYP-2::GFP (green) distribution patterns in late pachytene nuclei. Scale bar, 10 μm. (**D**) Quantification of SYP-2::GFP distribution patterns after 1,6-hexanediol treatment as performed in **C**. Numbers of gonads analyzed are indicated. Asterisks indicate statistical significance between different genotypes under the same condition: *P < 0.05, ***P < 0.001, by Chi-square test.

Although *syp-5* mutants did not display obvious differences in synapsis defects at 20°C and 25°C, more severe defects in CO regulation were present at the higher temperature. Given that synapsis and CO designation are intimately coupled, we speculated that alterations in SC internal properties may account for the abnormal CO regulation in the mutants. To test this, we examined SC properties through different strategies. HTP-3 is a core axial component required for SC central region proteins to be assembled along the lengths of chromosomes during meiotic prophase. In *htp-3* mutants, SC central region proteins form polycomplexes (Goodyer et al., 2008; Severson et al., 2009). Interestingly, polycomplexes formed in *htp-3* mutants, but not heat-induced SC aggregates, exhibit similar properties to the normally assembled SC (Rog et al., 2017). Indeed, *htp-3* RNAi resulted in polycomplex formation in pachytene nuclei as revealed by the formation of bright SYP-2::GFP foci. However, no bright SYP-2::GFP focus was observed in the *syp-5* mutant background after HTP-3 depletion by RNAi at 20°C or 25°C, and diffused SYP-2::GFP signal was observed instead (Figure 7A and B). We also examined SC aggregate formation induced by high temperature at 27°C, and similar frequencies of aggregate formation were observed in the wild-type and *syp-5* mutants (Supplementary Figure S4). These analyses suggest that SYP-5 is critical for the SC to maintain the normal properties that allow efficient aggregation in the absence of HTP-3 and that extreme temperatures can cause SC aggregate formation independent of SYP-5.

We also tested SC sensitivity to 1,6-hexanediol, a reagent that can disrupt SC structure in multiple organisms within a certain range of concentrations (Rog et al., 2017). When wild-type *C. elegans* gonads were treated with 5% 1,6-hexanediol, different SC diffusion levels were observed between early and late pachytene stages, with more gonads showing partial or intact SC remaining in nuclei at late pachytene stage (Figure 7C and D). Different temperatures (20°C and 25°C) did not significantly affect the hexanediol sensitivity in wild-type gonads (Figure 7D). The different hexanediol sensitivity may suggest the different SC properties between early and late pachytene stages, which has been revealed by the work from Colaiácovo and Villeneuve labs by fluorescence recovery after photobleaching analysis (Nadarajan et al., 2017; Pattabiraman et al., 2017). When gonads of *syp-5* mutants were treated with the same concentration of 1,6-hexanediol, more gonads showed complete disruption of the SC in both early and late pachytene nuclei compared to the wild-type. Moreover, differences in SC diffusion pattern after 1,6-hexanediol treatment were also observed in the mutants between incubations at 20°C and 25°C, with more gonads showing cytoplasm diffused pattern at the higher temperature (Figure 7C and D). In conclusion, both polycomplex formation and 1,6-hexanediol sensitivity assays suggest the requirement of SYP-5 to maintain the normal properties of the SC, which may be critical for CO regulation at higher temperatures.

### CIE-removal mutants are temperature-sensitive

**Figure 8:**
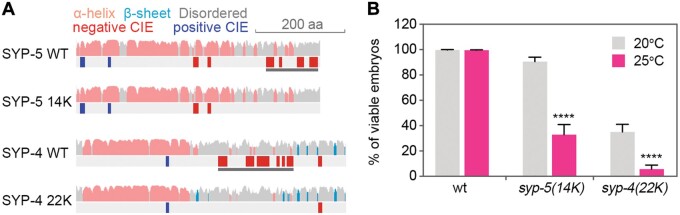
CIEs are required for meiosis thermotolerance. (**A**) Schematic diagrams show the presence of CIEs within wild-type or mutated SYP-5 and SYP-4. *syp-4(22K)* and *syp-5(14K)* point mutations resulted in the removal of C-terminal CIEs within the indicated gray bar regions (Zhang et al., 2020). (**B**) Analysis of progeny viability of the indicated genotypes at 20°C or 25°C. Error bars indicate standard error of the mean. Asterisks indicate statistical differences between different temperatures (****P < 10^−6^, by the two-tailed Mann–Whitney test, 95% confidence interval).

Our previous study showed that CIEs are abundant within SC proteins. Compared to SYP-6, SYP-5 contains more abundant CIEs at the C-terminal disordered region. Moreover, CIE removal by point mutations in SYP-4 and SYP-5 results in defective SC assembly and CO regulation (Figure 8A; Zhang et al., 2020). To examine whether CIEs in SYP-5 were required for meiotic thermotolerance, we analyzed the thermosensitivity of *syp-5(14K)* mutants, in which the C-terminal CIE cluster on SYP-5 is removed and all other SYP proteins are still present. Indeed, there was a significantly reduced percentage of viable embryos at the elevated temperature (Figure 8B), suggesting that CIEs in SYP-5 are critical for meiotic thermotolerance in *C. elegans*. To examine whether CIEs in other SYP proteins might also be critical for thermotolerance, we analyzed *syp-4(22K)* mutants with an absence of CIE cluster. There was also a significantly reduced percentage of viable progeny at 25°C compared to 20°C in this mutant (Figure 8B). These analyses suggest that CIEs in *C. elegans* SC proteins are critical for meiotic thermotolerance.

CIEs are not only present in *C. elegans* SC proteins but also in SC components of other organisms including humans (Zhang et al., 2020). Mutations that alter CIE presence may alter SC properties and thermotolerance. By analyzing human variants that cause missense mutations, we identified a group of variants that alter CIE presence within SC components including C14ORF39/SIX6OS1, SYCE1, SYCE2, and SYCP1 (Supplementary Figure S5). Further research on the effect of CIE removal on meiotic thermotolerance in mammals is required to understand its significance in human reproductive health.

## Discussion

Unravelling the underlying molecular mechanisms of meiotic thermotolerance is important for our understanding of evolution, breeding, genetic manipulation of meiosis to resist temperature extremes, and human reproductive health. Using the nematode *C. elegans*, here, we identified SC central region protein SYP-5 as a critical factor in meiotic thermotolerance.

Proper assembly of the SC complex is required for inter-homologous CO formation. By analyzing *syp-5* mutants, we observed increased frequencies of abnormal CO formation, but no apparent changes in SC assembly and disassembly at the examined elevated temperature (25°C), suggesting that CO regulation is more sensitive to temperature changes than SC assembly and disassembly. Although the SC structure is similar at different temperatures, its internal properties might be different, which may contribute to the defects in CO regulation seen at higher temperatures. Indeed, polycomplex formation and hexanediol sensitivity analysis revealed that the SC in *syp-5* mutants exhibited different properties compared to the wild-type. By performing time course analysis of RAD-51 focus formation in the germline, we did not observe a change in RAD-51 foci at different temperatures, suggesting that SPO-11-mediated DSB formation and early steps of meiotic recombination might not be significantly affected by the elevated temperature in *syp-5* mutants, highlighting the special role of the SC in sensing temperature changes. The sensitivity of CO regulation to temperature changes may benefit organism adaptation to environmental changes by increasing the shuffling of genes in the population while maintaining some reproduction capacity.

SC central region and axial components form aggregates at extreme temperatures across species (Morgan et al., 2017). Collapse of meiotic structures is accompanied with a failure in meiosis, and the ability to adapt is thus lost. At such extreme temperatures, collapse of meiosis structures might be an outcome of protein misfolding and aggregation, which are well-known effects of extreme temperatures affecting a large range of biological processes (Hartl et al., 2011; Powers and Balch, 2013). Axial and SC proteins usually contain sequences of disordered regions, which may render them more prone to aggregation (Gao and Colaiácovo, 2018). At 27°C, we observed SC aggregate formation in both the wild-type and *syp-5* mutants, which may represent a collapse of the SC that is independent of the normal regulatory function of SYP-5.

In analyzing a group of meiosis-defective mutants, we also identified *zim-3* and *lab-1* as thermosensitive mutants. Interestingly, all the identified thermosensitive mutants exhibit synapsis defects, which raises the possibility that intact SC is critical for thermotolerance. However, this is challenged by the fact that no thermosensitivity was observed for other two synapsis-defective mutants *him-8* and *zim-2*. While HIM-8 and ZIM-2 mediate the pairing of only one of the six pairs of chromosomes, ZIM-3 mediates that of two pairs of chromosomes (Phillips and Dernburg, 2006). One possibility is that the SC may overload onto the normally paired chromosomes in *zim-3* mutants, resulting in abnormal CO regulation at the elevated temperature. Chromosome remodeling involves protein relocalization along the chromosome arms during late meiotic prophase, as asymmetric SC disassembly takes place and bivalent cruciform structures form (Martinez-Perez et al., 2008). LAB-1 localizes to the bivalent long-arms and protects long-arm sister chromatid cohesion during late meiotic prophase (de Carvalho et al., 2008). Moreover, LAB-1 also plays critical roles during early prophase and its depletion affects CO regulation (Tzur et al., 2012), it is possible that the temperature sensitivity of *lab-1* mutants is associated with the abnormal CO regulation. However, it remains to be investigated whether depletion of LAB-1 causes a change in SC properties.

Mutations in several genes have been shown to have temperature-specific meiotic defects in *C. elegans*, including *ife-2*, *pgl-1*, and *pch-2* (Song et al., 2010; Bilgir et al., 2013; Deshong et al., 2014). Interestingly, defects in SC formation and/or CO regulation were also reported for these mutants, although the mechanisms underlying their impacts are likely different. IFE-2 is proposed to promote the translation of the ZMM protein MSH-4/5 at high temperatures to stabilize CO maturation (Song et al., 2010). PGL-1 is required for proper SC formation/maintenance at the higher temperature potentially by inhibiting premature meiotic gene expression or by altering heat shock protein levels (Bilgir et al., 2013). PCH-2 localizes to the SC during early meiotic prophase and is proposed to coordinate pairing, synapsis, and recombination to promote CO assurance (Deshong et al., 2014). These findings further suggest that the SC and other factors controlling CO formation play critical roles in regulating meiotic thermotolerance. It would be interesting to examine whether these mutations affect SC properties.

Meiosis takes place at a wide range of temperatures in different organisms. In plants, the ability to sense and respond to environmental factors in a plastic manner is of significant importance due to their immobility. In fact, plants have evolved to have a wider range of permissive temperatures for meiosis compared to animals (Bomblies et al., 2015). The SC functions as the core structure in controlling CO formation. SC stability may be different in different organisms to allow the SC to function at different temperatures. Several interactions may be involved in SC formation including hydrophobic interactions and charge-mediated interactions. The number and type of interactions present in the SC of an organism may thus define the SC properties and the functional temperature range.

The SC in *C. elegans* is highly dynamic and has liquid crystalline properties (Rog et al., 2017; Zhang et al., 2020). Such properties might be critical for signal transduction within the SC compartment and thus CO regulation (Zhang et al., 2018). We recently demonstrated the enrichment of CIEs within SC components and SC-recruiting proteins (Zhang et al., 2020). CIEs mediate weak interactions and may function as one of the major types of interaction within the SC, allowing it to be highly dynamic and creating a medium critical for signal transduction within the SC for CO interference. Removal of CIEs from components of the SC may thus alter its dynamic properties and signal transduction ability. The liquid crystalline property of the SC makes it a temperature-sensitive structure, and the alteration of CIEs within the SC may thus result in a change in optimal temperature range for meiosis.

It is worth noting that the SC central region might not be the only structure responsible for CO interference regulation. The chromosome axis has also been shown to mediate CO interference in yeast (Zhang et al., 2014). Interestingly, meiotic axial proteins also show the enrichment of CIEs, including Red1 and Hop1 in yeast (data not shown). Although axial components are not as dynamic as the central region, it would be intriguing to explore the role of the CIEs in axial proteins in CO interference and meiotic thermotolerance regulation. Moreover, CO regulation can be affected by DNA methylation, chromatin modifications, and RNA splicing during plant meiosis at altered culture temperatures (Modliszewski and Copenhaver, 2017). Genes involved in these biological processes are also potential regulators of meiotic thermotolerance in various organisms.

## Materials and methods

### C. elegans genetics

N2 Bristol was used as the wild-type strain, and all mutants used in this study were derived from the N2 background (Table 1). Worms were cultured at 20°C or 25°C on nematode growth medium plates.

### Plate phenotyping

To score egg viability and male progeny, L4 hermaphrodites were singled onto individual plates and their eggs were counted immediately after each laying period (every 24 h). Surviving progeny and males were scored when worms reached the adult stage.

### Immunofluorescence microscopy

All analyses were performed on 24 h post-L4 adults grown at 20°C or 25°C. Gonad dissection, fixation, immunostaining, and DAPI counterstaining were performed as previously described (Gao et al., 2016). The following primary antibodies were used: goat anti-SYP-1 (1:1000; Clemons et al., 2013), guinea pig anti-HTP-3 (1:500; Goodyer et al., 2008), rabbit anti-phospho-H3 (Ser10; 1:1000; Thermo Fisher Scientific), and rabbit anti-RAD-51 (1:10000; Novus Biologicals). Secondary antibodies used were donkey anti-goat-Cy3, donkey anti-rabbit-Cy3, and anti-guinea pig-Alexa Fluor 488 (all 1:200; Jackson ImmunoResearch Laboratories). Fluorescence microscope images were maximum-intensity projections through 3D data stacks of whole nuclei. Images were captured through whole nuclei at 200-nm intervals on a DeltaVision OMX microscope system with 60× (NA 1.42) objective lens using SoftWoRx software (Applied Precision) in conventional imaging mode and deconvolved using a conservative algorithm with 10 iterations. Adjustments of brightness and/or contrast were made in Adobe Photoshop. For each immunostain, at least three gonads were analyzed for each genotype.

**Table 1:** The strains used in this study.

Strain	Genetics
wild-type	N2
AV630	*meis8[pie-1p::gfp::cosa-1+unc-119(+)]* II
AV270	*him-3(me80)/nT1[qIs50]* (IV;V)
CA258	*zim-2(tm574)* IV
CA448	*unc-24(e138) zim-3(tm2303)* IV
CB1489	*him-8(e1489)* IV
CV2	*syp-3(ok758)* I*/hT2[qIs48]* (I;III)
CV6	*lab-1(tm1791)* I*/hT2[qIs48]* (I;III)
FM125	*unc-119(ed3)*;*ruls57[pAZ147: pie-1/β-tubulin::GFP;unc-119(+)]*;*itls37[unc-119(+) pie-1::mCherry::H2B]*
MGC2	*syp-5(cac1)* I
MGC4	*syp-5(cac1)* I;*meis8[pie-1p::gfp::cosa-1+unc-119(+)]* II
MGC9	*syp-5(cac1)* I;*unc-119(ed3)*;*ruls57[pAZ147: pie-1/β-tubulin::GFP;unc-119(+)]*;*itls37[unc-119(+) pie-1::mCherry::H2B]*
MGC126	*syp-4(22K)(cac42)* I
MGC13	*syp-6(cac3)* I
MGC181	*syp-5(cac43)* I;*meis8[pie-1p::gfp::cosa-1+unc-119(+)]* II
VC257	*htp-1(gk150)* IV

### Time course analysis of RAD-51 and GFP::COSA-1 foci

Quantification of RAD-51 foci along the germline was performed as described before (Gao et al., 2015). The germline of age-matched (24 h post-L4) worms were immunostained for RAD-51. At least four gonads were quantified per genotype per condition. For GFP::COSA-1 focus quantification, gonads dissected from worms expressing GFP::COSA-1 were fixed and counterstained with DAPI. 3D data stacks of whole nucleus images were captured on a DeltaVision OMX microscope, and GFP::COSA-1 foci were counted with SoftWoRx software.

### In vivo meiotic spindle examination

To visualize meiotic spindles, a strain expressing H2B::mCherry and β-tubulin::GFP (FM125 strain) was used. Worms were collected in 1.5 ml Eppendorf tubes, and fixed with −20°C methanol for 1 min and followed by 4% paraformaldehyde for 25 min. After a PBST (phosphate-buffered saline + 0.1% Tween 20) wash, DAPI (1 μg/ml; Sigma Aldrich) was used to stain DNA, and the worms were mounted with Vectashield (Vector Laboratories Ltd) mounting medium on slides. Meiotic spindles were examined with a Nikon TS2-FL microscope.

### Hexanediol sensitivity assay

To analyze SC sensitivity to 1,6-hexanediol at different temperatures, worms grown at 20°C or 25°C were dissected in Leibovitz’s L-15 medium (Biological Industries) supplied with 10% fetal bovine serum (Biological Industries) and 0.01% Tween 20 (complete L-15 medium). Intact gonads were transferred to 1 ml complete L-15 medium in 1.5 ml tubes incubated in 20°C or 25°C water bath for 5 min. After the settlement of the gonads, the supernatant was removed and 1 ml complete L-15 medium containing 5% 1,6-hexanediol was added. After 5 min incubation in 20°C or 25°C water bath, the supernatant was removed and the gonads were fixed with 1 ml 4% paraformaldehyde in PBS for 30 min. After two washes with PBST, gonads were stained with DAPI and mounted on slides with coverslips. Images were captured with a Nikon TS2-FL microscope.

### SC polycomplex formation assay

Feeding RNAi was performed for HTP-3 depletion in order to assess SC polycomplex formation. To construct the *htp-3* feeding RNAi clone, a segment of *htp-3* last exon was PCR-amplified with a pair of primers (forward primer: 5ʹ-ctcgagggggggcccggtaccgatccatcaggtgccgctc-3ʹ; reverse primer: 5ʹ-ctatagggcgaattgggtaccgtttcctcttctacttggcatgttc-3ʹ) and inserted into the pL4440 feeding vector at the *Kpn*I site. The constructed plasmids were transferred into HT115 bacteria, and bacteria carrying the empty pL4440 vector was used as control. Preparation of RNAi plates was performed as previously described (Gao et al., 2015). L4 worms were transferred to the RNAi plates and F1 progeny were examined for polycomplex formation.

### Bioinformatics analysis of protein sequence features and human variants

Protein secondary structure prediction was performed with Porter 5 (Torrisi et al., 2019). CIE scanning was performed as described in our previous work (Zhang et al., 2020). Variants present in human SC components were extracted from the Genome Aggregation Database (gnomad.broadinstitute.org; gnomAD v2.1.1). Only missense mutations were analyzed for their impact on CIE formation. Other types of mutations including in-frame deletion, frameshift, stop gained, stop lost, and splice region mutations were not included in our analysis.

## Supplementary material

Supplementary material is available at *Journal of Molecular Cell Biology* online.

## Supplementary Material

mjab035_Supplementary_Data
