# Supplementary material for: SYP-5 regulates meiotic thermotolerance in Caenorhabditis elegans
Source: J Mol Cell Biol. 2021 Jun 3;13(9):662–75. doi: 10.1093/jmcb/mjab035 (PMC8648394; doi:10.1093/jmcb/mjab035)
Supplement: mjab035_Supplementary_Data [file mjab035_supplementary_data.pdf]

Supplementary material

SYP-5 regulates meiotic thermotolerance in *Caenorhabditis elegans*

Yuanyuan Liu†, Qiuchen Zhao†, Hui Nie†, Fengguo Zhang†, Tingting Fu, Zhenguo Zhang, Feifei Qi, Ruoxi Wang, Jun Zhou, and Jinmin Gao*

Institute of Biomedical Sciences, College of Life Sciences, Key Laboratory of Animal Resistance Biology of Shandong Province, Shandong Normal University, Jinan 250014, China

† These authors contributed equally to this work.

* Correspondence to: Jinmin Gao, E-mail: jinmingao@sdnu.edu.cn; Tel: +86-531-8618-2518

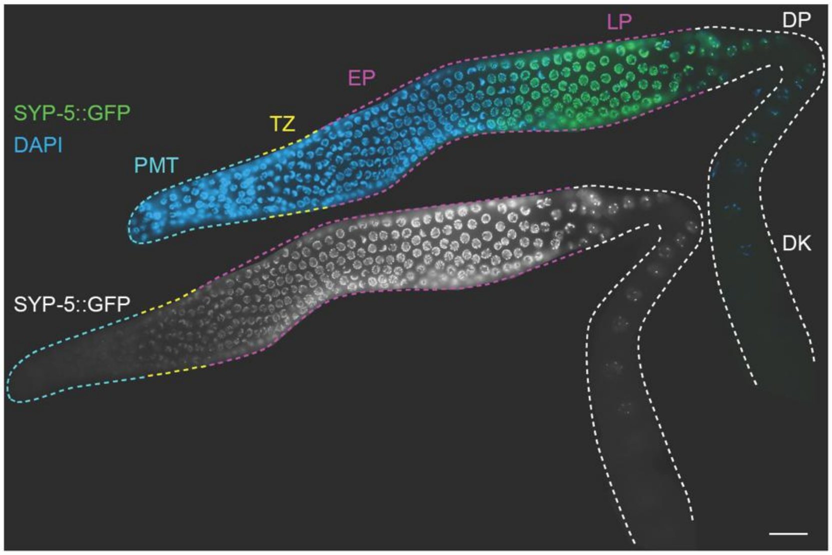

Supplementary Figure S1. SYP-5::GFP expression in the adult hermaphrodite germline. Gonads were dissected from young adult worms (24 h post-L4) expressing SYP-5::GFP (green) and fixed. Chromatin was stained with DAPI (blue). Dashed lines depict the outlines of the gonads. PMT, premeiotic tip; TZ, transition zone; EP, early pachytene; LP, late pachytene; DP, diplotene; DK, diakinesis. Bar, 20 µm.

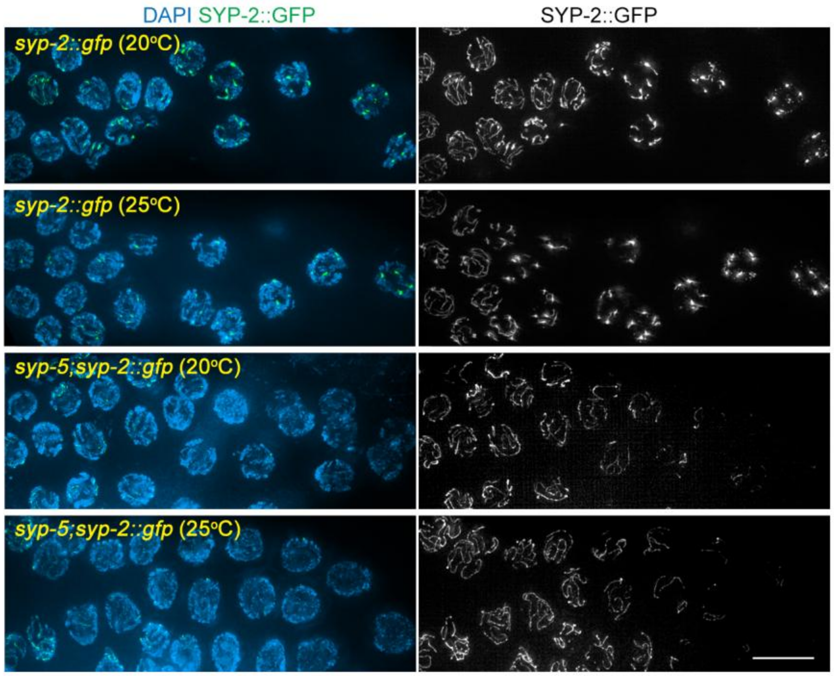

Normal SC maintemance (%)	Premature SC disassembly upon pachytene exit (%)	# of gonads
100	0	32
100	0	28
0	100	45
0	100	33

Supplementary Figure S2. Defects in SC disassembly are not temperature-sensitive in *syp-5* mutants. Gonads dissected from the indicated genotypes were stained with DAPI to visualize chromatin (blue). Kinetics of SC disassembly, as visualized by SYP-2::GFP (green), are similar at the examined culture temperatures in the indicated genotypes. Bar, 10 μm.

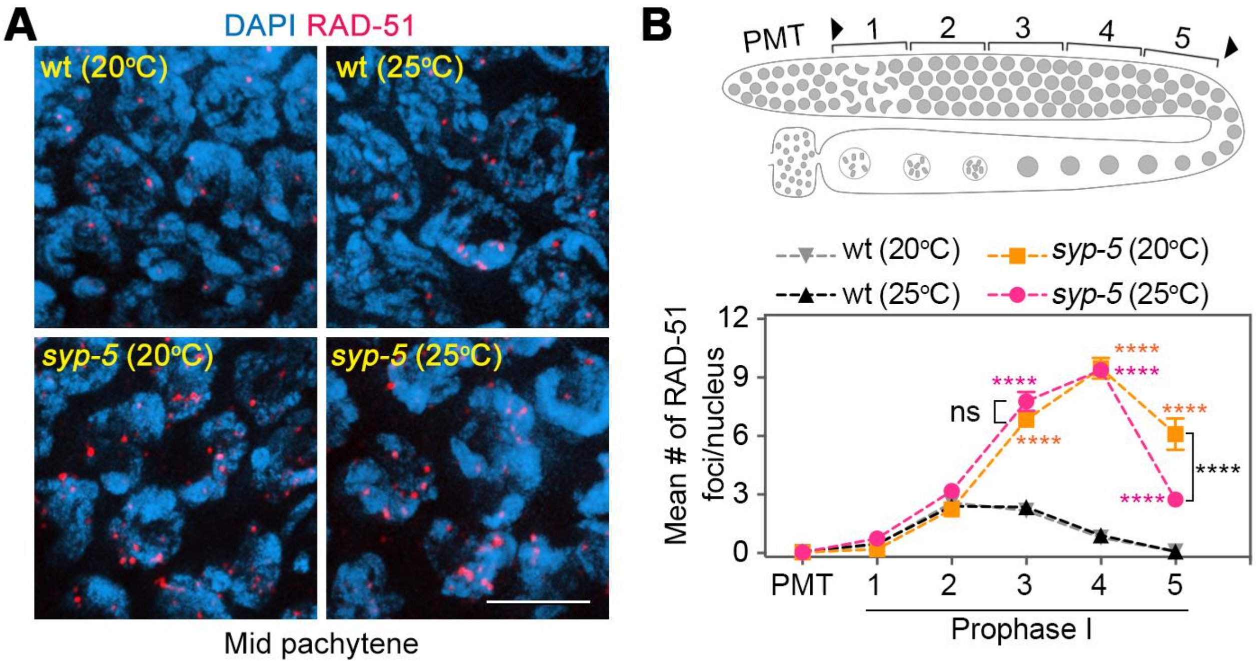

Supplementary Figure S3. Time course anlysis of DSB levels at different temperatures. (**A**) RAD-51 (red) immunostaining in mid-pachytene nuclei of wildtype and *syp-5* mutant worms grown at 20°C or 25°C. Chromatin was stained with DAPI (blue). Bar, 5 μm. (**B**) Quantification of RAD-51 foci in the germline. Top: Diagram of a *C. elegans* germline showing the position of the zones scored for RAD-51 foci in prophase I. PMT, premeiotic tip; zones 1-5 correspond to: transition zone (1), early (2), mid (3-4), and late (5) pachytene stages. Bottom: Levels of RAD-51 foci observed along the germline in the indicated genotypes and conditions. Colored asterisks indicate statistical significance between different genotypes under the same conditions: **** $P<0.0001$, by the two-tailed unpaired *t* test. ns, not statistically significant.

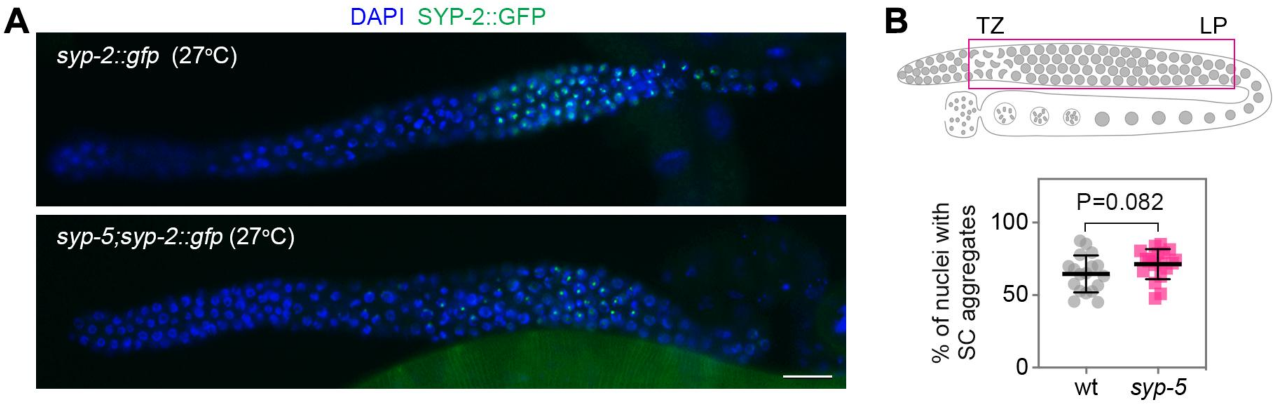

Supplementary Figure S4. Heat-induced SC aggregate formation. (**A**) L4 worms of the indicated genotypes were grown at 20°C for 12 hours and then incubated at 27°C for another 12 hours before dissection. Dissected gonads expressing SYP-2::GFP (green) were fixed and counterstained with DAPI (blue). Bar, 20 μm. (**B**) Quantification of SC aggregate formation after high temperature incubation as performed in A. Nuclei from transition zone (TZ) to late pachytene (LP) were scored. Statistical analysis was performed by the two-tailed unpaired *t* test.

A

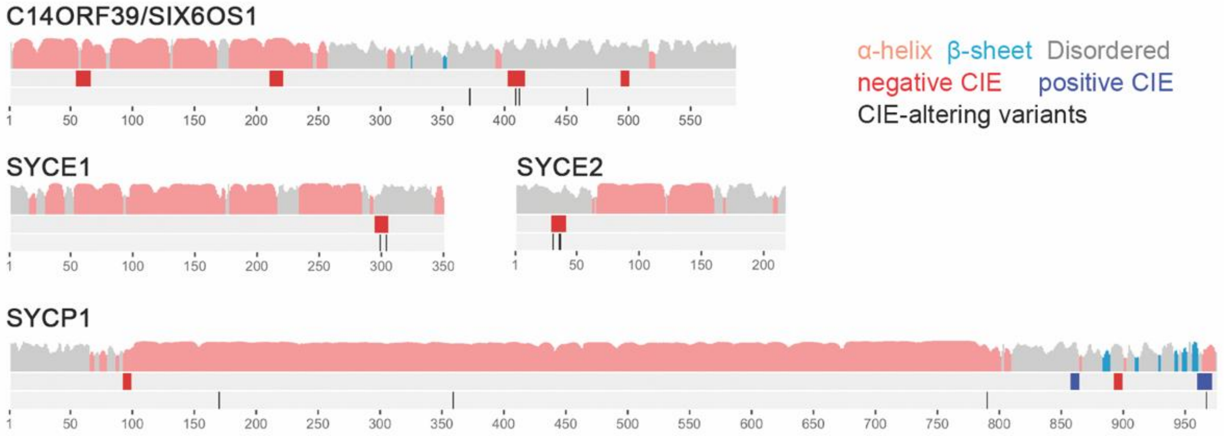

B

Protein	CIE enhanced	CIE impaired
C14ORF39	Lys372Glu<br>Lys476Glu	Asp409Gly<br>Glu412Gly
SYCE1		Glu299Ala<br>Gly304Arg
SYCE2		Glu31Lys<br>Glu36Lys<br>Glu37Lys
SYCP1	Lys170Glu<br>Lys359Gln<br>Lys359Ile<br>Lys790Lys	Lys967Glu<br>Lys967Asn

Supplementary Figure S5. Variants in humans alter CIE presence in SC proteins. Secondary structure and CIE segments are illustrated as in **Figure 8A**. Missense variants that alter CIE segment formation are indicated by black vertical lines (**A**) and summarized in the table (**B**). Human variants were sourced from the Gnome Aggregation Database (gnomad.broadinstitute.org).
